# The *Malat1* long non-coding RNA is upregulated by signalling through the PERK axis of unfolded protein response during flavivirus infection

**DOI:** 10.1038/srep17794

**Published:** 2015-12-04

**Authors:** Sankar Bhattacharyya, Sudhanshu Vrati

**Affiliations:** 1 Vaccine and Infectious Disease Research Centre, Translational Health Science and Technology Institute, NCR Biotech Science Cluster, 3rd Milestone, Faridabad – Gurgaon Expressway, Faridabad 121001, Haryana, India

## Abstract

Flavivirus infection causes host cell death by initiation of an **u**nfolded **p**rotein **r**esponse (UPR). UPR is initiated following activation of three ER-membrane resident sensors, PERK, IRE1α and ATF6, which are otherwise kept inactive through association with the ER-chaperone GRP78. Activation precedes cellular and molecular changes that act to restore homeostasis but might eventually initiate apoptosis. These changes involve influencing function of multiple genes by either transcriptional or post-transcriptional or post-translational mechanisms. Transcriptional control includes expression of transcription factor cascades, which influence cognate gene expression. *Malat1* is a long non-coding RNA which is over-expressed in many human oncogenic tissues and regulates cell cycle and survival. In this report, for the first time we show activation of *Malat1* following infection by two flaviviruses, both of which activate the UPR in host cells. The temporal kinetics of expression was restricted to later time points. Further, *Malat1* was also activated by pharmacological inducer of UPR, to a similar degree. Using drugs that specifically inhibit or activate the PERK or IRE1α sensors, we demonstrate that signalling through the PERK axis activates this expression, through a transcriptional mechanism. To our knowledge, this is the first report of an UPR pathway regulating the expression of an lncRNA.

Flaviviridae constitutes an important group of human pathogenic virus responsible for extensive death and debilitation in different parts of the world^1^. In addition to innate anti-viral immune pathways the infection following multiple flaviviruses activates an unfolded protein response (UPR) through saturation of the protein folding capacity in host cell endoplasmic reticulum (ER)^2^,^3^,^4^. The induction of UPR has been suggested as the principal cause behind the apoptotic cell death observed in infected cells. UPR is induced following infection by a wide variety of viruses, many of which have evolved to regulate the downstream signalling^5^.

The primary objective of the cellular changes observed during an UPR is to restore homeostasis, failing which the cell is committed to an apoptotic death. Unfolded/malfolded proteins that accumulate in the ER lumen form stable complexes with the ER chaperone HSPA5/Bip/GRP78. Under homeostatic conditions, GRP78 remains associated with the ER-lumen resident domain of three ER-membrane resident trans-membrane protein sensors, namely **P**KR-like **ER K**inase (PERK), **I**nositol-**r**esponsive **e**nzyme 1 (IRE1) and **A**ctivating **t**ranscription **f**actor 6 (ATF6). A continued association with GRP78 molecules maintains these sensors in a dormant state. The accumulated unfolded proteins compete with these sensors for binding to GRP78 molecules, thereby activating them. The activated sensors transduce the signal to different parts of the cell initiating the UPR. As part of UPR multiple transcription factors (TFs) are either activated or synthesized *de novo*, a few of them initiating a cascade of TF expression^6^. Additionally, the load of nascent proteins in the ER-lumen for folding is decreased through an inhibition of the ER-associated mRNA translation. This is achieved through phosphorylation of the translation initiation factor eIF2α by PERK and degradation of mRNAs that are associated with ER-membrane bound ribosomes by IRE1α^6^,^7^. In addition to inhibition of translation, activation of PERK also stimulates the transcriptional activity of NFE2L2^8^,^9^. The phosphorylation of eIF2α, attenuates rate of translation initiation on most mRNAs, but increases that for *Atf4*, which codes for the transcription factor (TF) ATF4^10^. One of the known target genes activated by ATF4, *Chop* or *Ddit3*, also codes for a TF with unique target genes^11^. However, in addition to the PERK axis, expression of *Ddit3* is also influenced through the activity of other UPR axes^12^. The over-expression of DDIT3 in the course of UPR which is induced by JEV infection, has been demonstrated to be of particular relevance with respect to the consequential apoptotic death of the infected cells^3^. In consonance, these three well characterized TFs (ATF4, NFE2L2 and DDIT3), drive the expression of multiple genes.

Long non-coding RNAs (LncRNA) from varied gene loci are increasingly being reported to function as crucial regulators of gene expression^13^. The mode of regulation can be either transcriptional through alteration of chromatin or post-transcriptional through influencing the choice of alternative splice sites or functioning as a sponge for specific microRNA(s)^13^,^14^,^15^,^16^. Recent reports have also indicated specific lncRNAs to have roles in cell survival^17^,^18^. The *Malat1* LncRNA has been reproducibly associated with aggressive carcinoma presenting a poor prognosis^19^,^20^. The physiological role of *Malat1* has been shown to involve cell growth, cell migration and cell cycle^19^,^21^. However, mice knock-out for *Malat1* did not exhibit any developmental aberrations^22^.

In this report we show *Malat1* to be upregulated by JEV infection of mouse neuroblastoma cells Neuro2a, presumably through an induction of UPR. As proof of that, *Malat1* was also upregulated by the pharmacological agent of UPR induction, thapsigargin or TG. Using different pharmacological drugs that either inhibit or activate specific UPR sensors, we present evidence that this upregulation is transcriptional and downstream of the signalling of the PERK axis of UPR.

## Methods

### Cell lines, virus infection and drug

Neuro2a and mouse embryonic fibroblast (MEF) cells were maintained in DMEM supplemented with 10% foetal- calf serum at 37 °C and 5% CO_2_. Japanese encephalitis virus (Vellore strain) and West Nile virus were grown in Porcine kidney cells (PS) or Vero cells as described earlier^23^. For the purpose of infection, virus stocks were diluted in DMEM-2% FCS and incubated with cells for 1 hour. At the end of infection, the inocula were discarded and complete growth medium overlaid on infected cells.

For treatment with different drugs complete media supplemented with 1μM of Thapsigargin (Sigma) either alone or supplemented with DMSO (appropriate concentration) or 150 nM of PERKi (Millipore) or 50μM of STF083010 (Sigma) or 1μg/ml of Actinomycin-D (Sigma), was overlaid on cells and incubated for the time indicated. For PERK activation cells were treated with complete media supplemented with 10μM CCT020312 (Millipore) for the indicated time.

### RNA isolation and real time PCR

Total RNA was extracted with Trizol reagent and purified using Qiagen RNeasy columns with in-column DNase digestion according to manufacturer’s protocol. The RNA was reverse-transcribed using 50 ng of random hexamers and Improm-II reverse transcriptase (Promega) according to manufacturer’s instructions. The cDNA was used for qPCR using 2x SYBR-green mix (ABI) in an ABI7500-Fast Real-time PCR machine. The primers used for qPCR are as follows: *Malat1* F (5′-AAGCAAGCAGTATTGTATCG-3′), *Malat1* R (5′-AGATGTTAAAACAAGCCCAG-3′); *Gapdh* F (5′-CGTCCCGTAGACAAAATGGT-3′), *Gapdh* R (5′-TTGATGGCAACAATCTCCAC-3′); *Ppp1r15a* F(5′-GAGGGACGCCCACAACTTC-3′), *Ppp1r15a* R (5′-TTACCAGAGACAGGGGTAGGT-3′); *Ddit3* F (5′-GTCAGTTATCTTGAGCCTAACACG-3′), *Ddit3* R (5′-TGTGGTGGTGTATGAAGATGC-3′); *Dnajc3* F (5′- GGCGCTGAGTGTGGAGTAAAT-3′), *Dnajc3* R (5′- GCGTGAAACTGTGATAAGGCG-3′); *St3gal5* F (5′-ATGCCAAGTGAGTTCACCTCT-3′), *St3gal5* R (5′-ACTCCAAATGCAACCAACGTG-3′).

### Immunoblot

Neuro2a cells treated with different drugs were washed with phosphate buffered saline and the total protein extracted using Cellytic lysis reagent (Sigma) supplemented with complete protease inhibitor cocktail (Roche). The protein concentration in all extracts were estimated using Bradford reagent (Biorad) and equal quantity of protein resolved using SDS-8%PAGE followed by immunoblotting. The immunoblot for total PERK was performed using anti-PERK antibody (C33E10; Cell Signalling) and that for phosphorylated-PERK using anti-phospho PERK antibody (16F8; Cell Signalling) according to manufacturer’s instructions. The immunoblots were visualized using western blotting luminol reagent (Santa Cruz).

## Results

### *Malat1* is overexpressed upon JEV and WNV infection

**Figure 1:**
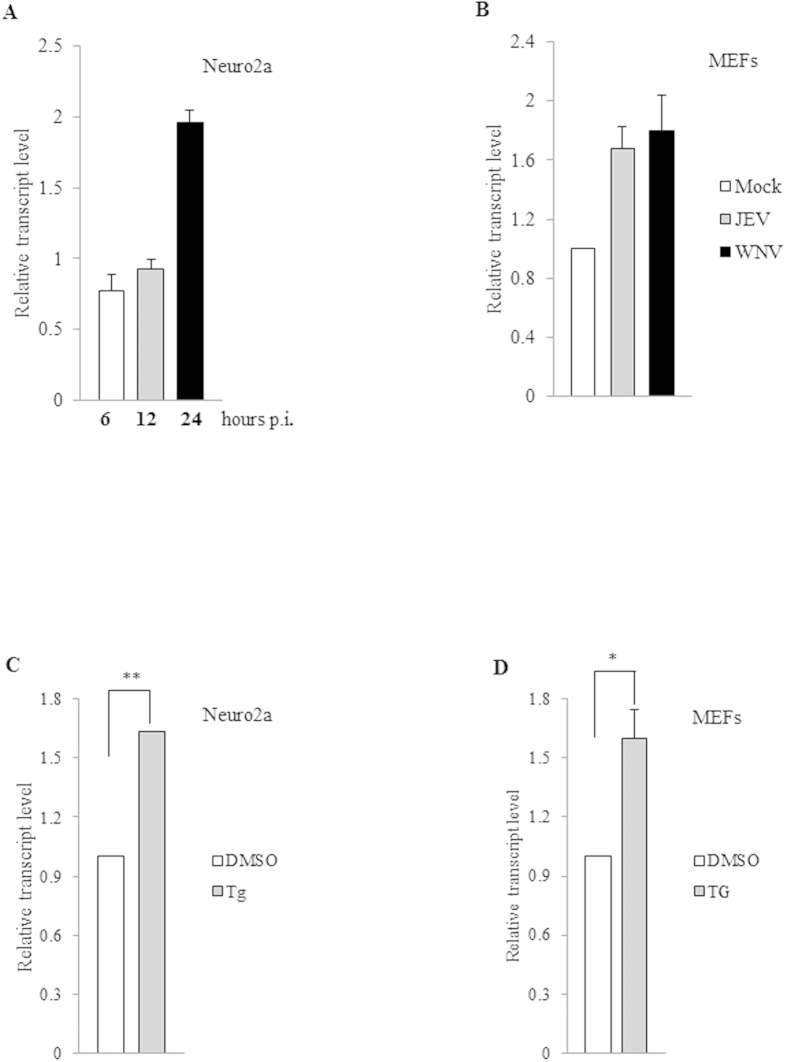
Upregulation of *Malat1* transcript level by flavivirus infection and UPR-inducing drug thapsigargin. (**A**) Total RNA was isolated from either mock- or JEV-infected (MOI = 5) Neuro2a cells at 6, 12 or 24 h post-infection. The RNA was reverse-transcribed and used for qPCR estimation of *Malat1* transcripts normalized to that of *Gapdh.* For each time point, the relative transcript level in JEV-infected cells was compared to that in mock-infected control and plotted as the fold-change. The time points of RNA extraction post-infection has been indicated from each bar **(B)** Total RNA was isolated from either mock- or JEV- or WNV-infected MEFs cells at 24 h post-infection. The RNA was reverse-transcribed and used for qPCR estimation of *Malat1* transcripts normalized to that of *Gapdh.* The relative transcript level in mock-infected cells was arbitrarily taken as 1 (white bar) and that in JEV-(grey bar) or WNV-infected cells (black bar) represented as fold-change over it. (**C,D)** Total RNA was isolated from DMSO- or TG-treated Neuro2a or MEF cells at 6 h after addition of drug. The RNA was reverse-transcribed and used for qPCR estimation of *Malat1* transcripts normalized to that of *Gapdh.* The relative transcript level in DMSO-treated cells was arbitrarily taken as 1 (white bar) and that in TG-treated cells (grey bar) represented as fold-change over it. All experiments were done at least two times in triplicates and the error bars represent the standard deviation. (** and * signifies p < 0.01 and p < 0.05 respectively).

A study of the alterations in transcriptome in Neuro2a cells upon JEV infection showed a moderate but statistically significant (P < 0.01) over-expression of the *Malat1* long non-coding RNA, which was validated by real-time PCR analysis (data not shown). Further, a study of the temporal kinetics of this overexpression showed the activation to ensue between 12 and 24 h post-infection (p.i.) (Fig. 1, panel A). In addition to Neuro2a infected by JEV, mouse embryonic fibroblasts (MEFs) infected with either JEV or West Nile virus (WNV), a close relative of JEV, also showed statistically significant augmentation of *Malat1* transcript level (Fig. 1, panel B). The host cell responses that have been characterized following flavivirus infection include innate anti-viral response and unfolded protein response. In general innate immune anti-viral responses are early, while gene expression changes that follow UPR induction in flavivirus infected cells, has been shown to be induced about 12 h p.i^4^. Since the upregulation of *Malat1* was observed between 12–24 h p.i., we presumed this gene activation to be a part of the UPR, which is activated in Neuro2a cells following JEV infection. In accordance with such a supposition, we observed a similar degree of upregulation of the *Malat1* transcript in cells that were treated with thapsigargin (TG), a pharmacological inducer of UPR. (Fig. 1, panel C). In order to assess the generality of this observation, the effect of TG-treatment in regulating *Malat1* level was tested on three other cell lines, two of them being of mouse origin (Mouse embryonic fibroblast cells or MEFs and the mouse microglial cell line BV2) and one of human origin (Human embryonic kidney cells or HEKs). Interestingly, although TG-treatment showed a similar effect on the *Malat1* level in MEFs, it did not induce *Malat1* level in either BV2 or HEKs (Fig. 1, panel D and data not shown). Expectedly, JEV infection of BV2 also did not induce augmentation of *Malat1* level (data not shown).

### *Malat1* is over-expressed as part of the UPR downstream of the PERK sensor

**Figure 2:**
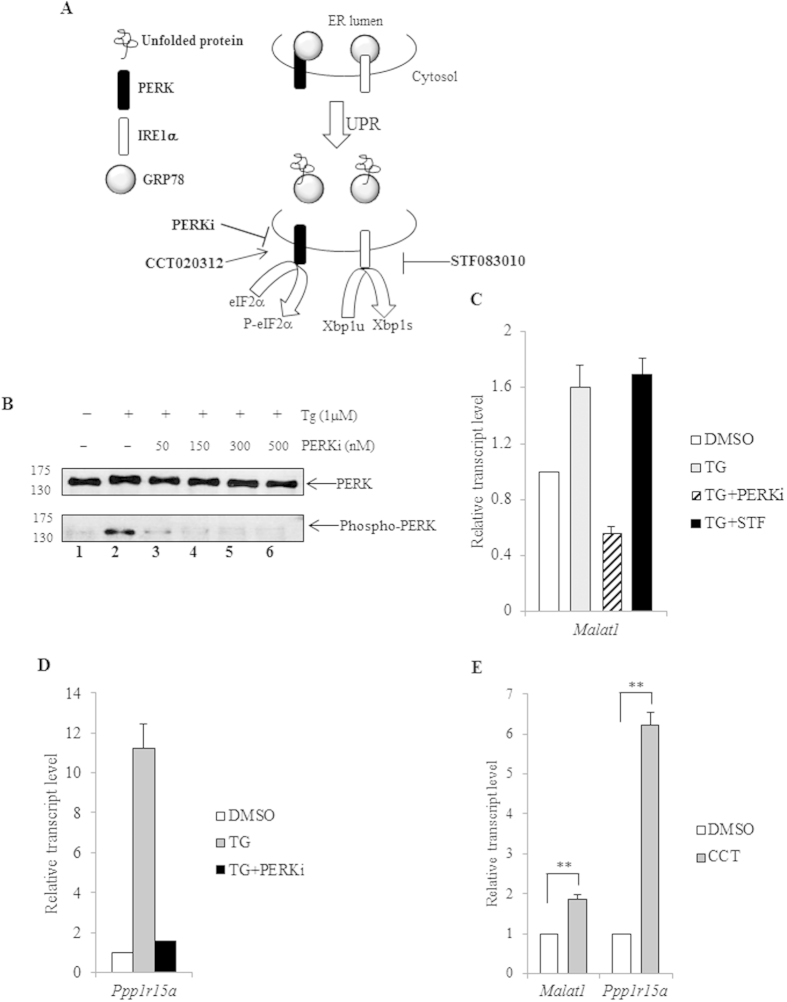
*Malat1* upregulation during UPR is in response to activation of PERK. (**A**) Schematic representation of the activation of PERK and IRE1α sensors during the UPR. The biochemical steps that are regulated by the UPR-influencing drugs PERKi, STF083010 and CCT020312 used in subsequent experiments are indicated. (**B**) Equal quantity of total protein isolated from Neuro2a cells which were untreated (lane 1) or treated with either TG alone (lane 2) or TG supplemented with increasing concentration of PERKi (lanes 3–6), was resolved in SDS-PAGE and immunoblotted using either anti-PERK or anti-phospho-PERK antibody. The numbers on the left indicate the migration of relative protein molecular weight marker bands. (**C**) Total RNA was isolated from Neuro2a cells treated with either DMSO (white bar) or TG (grey bar) or TG supplemented with PERKi (hashed bar) or TG supplemented with STF083010 (black bar), at 6 h after addition of drugs. The total RNA was reverse-transcribed and used for qPCR estimation of *Malat1* transcript level, normalized to that of *Gapdh* mRNA. The relative transcript level in DMSO-treated cells was taken arbitrarily as 1 and that in others represented as fold-change over it. (**D**) Total RNA was isolated from Neuro2a cells treated with either DMSO (white bar) or TG (grey bar) or TG supplemented with PERKi (black bar), at 6 h after addition of drugs. The total RNA was reverse-transcribed and used for qPCR estimation of *Ppp1r15a* transcript level, normalized to that of *Gapdh* mRNA. The relative transcript level in DMSO-treated cells was taken arbitrarily as 1 and that in others represented as fold-change over it. (**E**) Total RNA was isolated from Neuro2a cells treated with either DMSO or CCT020312 at 6 h after addition of drugs. The total RNA was reverse-transcribed and used for qPCR of the respective gene transcripts, normalized to that of *Gapdh* mRNA. The transcript level in DMSO-treated cells was arbitrarily taken as 1 (white bar) and that from CCT020312-treated cells (grey bar) represented as fold-change over it. All experiments were done in triplicates and the error bars represent the standard deviation (** signifies p < 0.01).

The gene expression programme characteristic of UPR is driven by activation of three ER-resident sensors. The activation of PERK induces autophosphorylation followed by phosphorylation of eIF2α, which in turn induces a global inhibition on protein synthesis. This autophosphorylation of PERK, and thereby the subsequent steps, can be inhibited by PERKi (Fig. 2, panel A)^24^. A similar autophosphorylation is observed subsequent to stimulation of the IRE1α sensor^25^. Phosphorylation activates an incipient RNase activity in IRE1α which performs a unique cytoplasmic splicing of the transcript coding for XBP1U (*Xbp1u*) to produce the transcript that can code for the TF XBP1S (*Xbp1s*)^25^. The RNase activity of IRE1α can be inhibited by the specific inhibitor STF083010 (Fig. 2, panel A)^26^. Co-treatment of Neuro2a cells with TG and different concentration of PERKi showed a total inhibition of the TG-mediated PERK phosphorylation in Neuro2a by 150 nM of the inhibitor (Fig. 2, panel B, compare lanes 1, 2 and 4). Previously, we have demonstrated inhibition of IRE1α RNase activity upon addition of STF083010 to Neuro2a cells^27^. A pharmacological inhibition, of either of these pathways in TG-treated Neuro2a cells using the respective drugs, showed the signalling initiated by PERK but not that by IRE1α to be responsible for *Malat1* upregulation (Fig. 2, panel C). An inhibition of PERK activation by PERKi would suggest reduced synthesis of ATF4, which would be evident through a reduction in the transcription of its cognate target genes. As further proof of a block in signalling downstream of activated PERK upon addition of PERKi, we observed a reversal of TG-mediated over-expression of the ATF4 target gene *Ppp1r15a* (Fig. 2, panel D)^28^. It is possible to pharmacologically activate only the PERK sensor, without sensitizing the other, by treatment of cells with the drug CCT020312 (CCT)^29^. As further confirmation of the involvement of PERK signalling, activation of only the PERK axis by CCT showed increase in *Malat1* level (Fig. 2, panel E). As a proof of activation of the PERK axis we observed upregulation of the ATF4 target genes *Ppp1r15a* (Fig. 2, panel E)^28^.

### The increase in *Malat1* level is transcriptional

**Figure 3:**
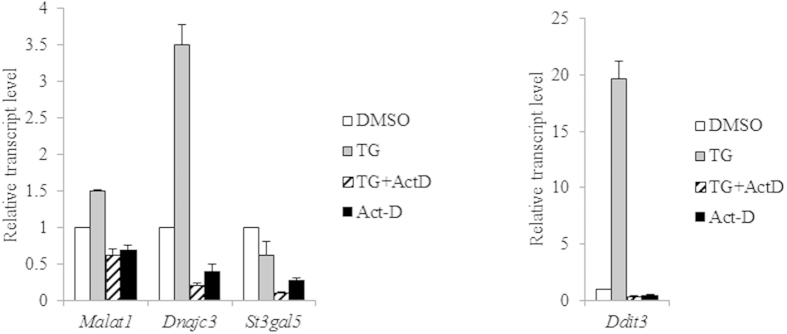
PERK activates transcription of *Malat1*. Total RNA was isolated from Neuro2a cells treated with DMSO or TG or TG supplemented with Actinomycin-D or Actinomycin-D alone, at 6 h after the addition of the drugs. The RNA was reverse-transcribed and used for qPCR estimation of transcripts from respective genes normalized to that of *Gapdh*. The relative transcript level in DMSO-treated cells (white bar) was arbitrarily taken as 1 and that in cells treated with TG (grey bar) or TG supplemented with Actinomycin-D (hashed bar) or Actinomycin-D alone (black bar) represented as fold-change over it. All experiments were done in triplicates. The error bars represent the standard deviation.

As part of an UPR, alterations in the expression of genes coding for both proteins and non-coding regulatory RNAs like microRNAs, is observed^30^. Further, *Malat1* has been shown to be amenable to negative regulation by three different miRNAs^31^,^32^. Therefore, to delineate the observed upregulation as either transcriptional or post-transcriptional, *Malat1* levels were compared between cells treated with either TG alone or TG in presence of Actinomycin-D (Act-D), a known inhibitor of RNA polymerase activity. The results showed that the upregulation of *Malat1* by TG-treatment can be reversed by Actinomycin-D (Fig. 3). As control, we observed similar reversal for transcripts coding DDIT3 and DNAJC3, which are respectively activated transcriptionally by the UPR-related transcription factors ATF4 and XBP1 (Fig. 3)^33^,^34^. Further, as noted earlier, treatment with TG in the presence of Act-D, led to an even higher repression of *St3gal5* transcript, which is degraded by the IRE1α as part of RIDD pathway of gene regulation (Fig. 3)^35^. A treatment of Neuro2a cells Act-D alone repressed *Malat1* level to those observed upon simultaneous addition of Act-D and TG, indicating a continuous transcription of the gene under basal conditions, which is augmented during UPR in a PERK dependent manner.

## Discussions

The gene expression changes in a virus-infected host cells can potentially have an antiviral or a proviral effect. The error-prone genome replication of RNA viruses provides molecular variants that can exploit a favourable change and occupy the respective niche, thus driving viral evolution^36^. We have previously shown how UPR-specific gene regulatory pathways can have a beneficial effect on JEV replication^27^. The infection induced UPR has also been implicated as the gene expression program that drives cell death during JEV infection, by signalling downstream of the PERK axis^3^. In this report we show *Malat1*, an lncRNA previously established to play a critical role in metastasis of cancer cells, to be upregulated in JEV-infected Neuro2a cells. Further, *Malat1* was activated by pharmacological induction of UPR, through signalling downstream of the PERK sensor. In a manner similar to many other genes that are activated by this sensor, the upregulation of *Malat1* was transcriptional. To our knowledge, this is the first report that shows the *Malat1* gene to be upregulated following activation of a specific ER-sensor during ER-stress.

*Malat1* upregulation has been associated with metastasis of cancer cells, in which it has been shown to promote migration/invasion, an effect which can be reversed by RNAi-mediated silencing of the transcript^32^,^37^. Recent reports have implicated hypoxia as a trigger for activating *Malat1* expression, a condition which has also been reported to activate PERK signalling^38^,^39^. In separate reports the TGFβ pathway, the c-fos and Sp1 transcription factor have been shown to promote *Malat1* expression^20^,^40^,^41^. Also, endogenous post-transcriptional regulation of *Malat1* transcript by miRNAs has been demonstrated^31^. An exogenous depletion of *Malat1* was demonstrated to induce cell cycle arrest in G2/M phase with concomitant augmentation of apoptosis^20^,^37^.

The functions of *Malat1* have been implicated to be principally in regulating gene expression in addition to controlling alternative splicing, in a manner which is dependent on localization of the transcript to nuclear speckles, although it is not required for the integrity of nuclear speckles *per se*^15^,^42^,^43^,^44^. An association of this lncRNA with pre-mRNAs at active transcription loci, which leads to modulation in the expression of cell cycle regulating transcription factor genes, has been demonstrated^14^,^21^,^45^. In a manner that is relevant to understanding any plausible significance of *Malat1* over-expression in virus infected cells, this lncRNA has been shown to not have any significantly influential role in expression of interferon induced genes^43^.

At the cellular level *Malat1* transcript promotes inflammatory response, possibly through activation of the PI3/Akt and ERK/MAPK pathways^46^,^47^,^48^. At the molecular level the *Malat1* transcript has been shown to function as a competing endogenous RNA by acting as a miRNA-sponge for miR-133^49^. Previously, we performed differential expression analysis for microRNAs in JEV-infected Neuro2a cells versus mock-infected controls. The results showed no detectable expression of miR-133 in this cell line, thus negating the possibility of such a regulatory role for *Malat1* (data not shown).

Reports from multiple groups suggest ER-stress signalling to modulate virus infection cycle. In fact, ER-stress has been shown to enhance the Interferon pathway of innate anti-viral immune response^50^. Activation of lncRNA genes in different model system of host-pathogen interactions has been demonstrated earlier. Interestingly, Saha and co-workers showed upregulation of certain lncRNAs in mouse brain following JEV infection, one of which was *Neat1* (referred to as VINC in the article)^51^. Enhanced expression of *Malat1* has also been reported from clinical samples infected with human papilloma virus (HPV)^52^. In addition to JEV infection, HIV infected cells also showed over-expression of *Neat1*, another lncRNA which is expressed from a gene loci very close to that of *Malat1*^53^. Further, *Neat1* expression has been shown to have an anti-viral role in suppressing HIV replication, although no role in JEV infection is known^53^. Since both *Neat1* and *Malat1* have been shown to localize to active transcribing loci in the chromatin, this might suggest a potential anti-viral role for *Malat1* as well^14^. However, the implication of *Malat1* over-expression for UPR in general and virus-induced UPR in particular is still not clear yet, and would need further investigation.

## Additional Information

**How to cite this article**: Bhattacharyya, S. and Vrati, S. The *Malat1* long non-coding RNA is upregulated by signalling through the PERK axis of unfolded protein response during flavivirus infection. *Sci. Rep.* **5**, 17794; doi: 10.1038/srep17794 (2015).

## References

[b1] Lindenbach Brett D., Charles H.-J. u. T. & Rice M. Flaviviridae: The Viruses and Their Replication (ed. Knipe D. M. & Howley P. M. ) (Lippincott-Raven, Philadelphia, 2007).

[b2] Medigeshi G. R. *et al.* West Nile virus infection activates the unfolded protein response, leading to CHOP induction and apoptosis. J Virol 81, 10849–10860 (2007). 17686866 10.1128/JVI.01151-07 PMC2045561

[b3] Su H. L., Liao C. L. & Lin Y. L. Japanese encephalitis virus infection initiates endoplasmic reticulum stress and an unfolded protein response. J Virol 76, 4162–4171 (2002). 11932381 10.1128/JVI.76.9.4162-4171.2002 PMC155064

[b4] Yu C. Y., Hsu Y. W., Liao C. L. & Lin Y. L. Flavivirus infection activates the XBP1 pathway of the unfolded protein response to cope with endoplasmic reticulum stress. J Virol 80, 11868–11880 (2006). 16987981 10.1128/JVI.00879-06 PMC1642612

[b5] Chan S. W. The unfolded protein response in virus infections. Front Microbiol 5, 518 (2014). 25324837 10.3389/fmicb.2014.00518 PMC4179733

[b6] Schroder M. & Kaufman R. J. ER stress and the unfolded protein response. Mutat Res 569, 29–63 (2005). 15603751 10.1016/j.mrfmmm.2004.06.056

[b7] Hollien J. & Weissman J. S. Decay of endoplasmic reticulum-localized mRNAs during the unfolded protein response. Science 313, 104–107 (2006). 16825573 10.1126/science.1129631

[b8] Cullinan S. B. *et al.* Nrf2 is a direct PERK substrate and effector of PERK-dependent cell survival. Mol Cell Biol 23, 7198–7209 (2003). 14517290 10.1128/MCB.23.20.7198-7209.2003 PMC230321

[b9] Harding H. P., Zhang Y. & Ron D. Protein translation and folding are coupled by an endoplasmic-reticulum-resident kinase. Nature 397, 271–274 (1999). 9930704 10.1038/16729

[b10] Harding H. P. *et al.* An integrated stress response regulates amino acid metabolism and resistance to oxidative stress. Mol Cell 11, 619–633 (2003). 12667446 10.1016/s1097-2765(03)00105-9

[b11] Fawcett T. W., Martindale J. L., Guyton K. Z., Hai T. & Holbrook N. J. Complexes containing activating transcription factor (ATF)/cAMP-responsive-element-binding protein (CREB) interact with the CCAAT/enhancer-binding protein (C/EBP)-ATF composite site to regulate Gadd153 expression during the stress response. Biochem J 339 (Pt 1), 135–141 (1999). 10085237 PMC1220137

[b12] Yoshida H. *et al.* ATF6 activated by proteolysis binds in the presence of NF-Y (CBF) directly to the cis-acting element responsible for the mammalian unfolded protein response. Mol Cell Biol 20, 6755–6767 (2000). 10958673 10.1128/mcb.20.18.6755-6767.2000 PMC86199

[b13] Mercer T. R., Dinger M. E. & Mattick J. S. Long non-coding RNAs: insights into functions. Nat Rev Genet 10, 155–159 (2009). 19188922 10.1038/nrg2521

[b14] West J. A. *et al.* The long noncoding RNAs NEAT1 and *MALAT1* bind active chromatin sites. Mol Cell 55, 791–802 (2014). 25155612 10.1016/j.molcel.2014.07.012 PMC4428586

[b15] Tripathi V. *et al.* The nuclear-retained noncoding RNA *MALAT1* regulates alternative splicing by modulating SR splicing factor phosphorylation. Mol Cell 39, 925–938 (2010). 20797886 10.1016/j.molcel.2010.08.011 PMC4158944

[b16] Xia T. *et al.* Long noncoding RNA associated-competing endogenous RNAs in gastric cancer. Sci Rep 4, 6088 (2014). 25124853 10.1038/srep06088 PMC4133709

[b17] Pickard M. R., Mourtada-Maarabouni M. & Williams G. T. Long non-coding RNA GAS5 regulates apoptosis in prostate cancer cell lines. Biochim Biophys Acta 1832, 1613–1623 (2013). 23676682 10.1016/j.bbadis.2013.05.005

[b18] Atmadibrata B. *et al.* The novel long noncoding RNA linc00467 promotes cell survival but is down-regulated by N-Myc. PLoS One 9, e88112 (2014). 24586304 10.1371/journal.pone.0088112 PMC3929584

[b19] Ji P. *et al.* MALAT-1, a novel noncoding RNA, and thymosin beta4 predict metastasis and survival in early-stage non-small cell lung cancer. Oncogene 22, 8031–8041 (2003). 12970751 10.1038/sj.onc.1206928

[b20] Hirata H. *et al.* Long Noncoding RNA *MALAT1* Promotes Aggressive Renal Cell Carcinoma through Ezh2 and Interacts with miR-205. Cancer Res 75, 1322–1331 (2015). 25600645 10.1158/0008-5472.CAN-14-2931 PMC5884967

[b21] Tripathi V. *et al.* Long noncoding RNA *MALAT1* controls cell cycle progression by regulating the expression of oncogenic transcription factor B-MYB. PLoS Genet 9, e1003368 (2013). 23555285 10.1371/journal.pgen.1003368 PMC3605280

[b22] Zhang B. *et al.* The lncRNA *Malat1* is dispensable for mouse development but its transcription plays a cis-regulatory role in the adult. Cell Rep 2, 111–123 (2012). 22840402 10.1016/j.celrep.2012.06.003 PMC3408587

[b23] Vrati S., Agarwal V., Malik P., Wani S. A. & Saini M. Molecular characterization of an Indian isolate of Japanese encephalitis virus that shows an extended lag phase during growth. J Gen Virol 80 (Pt 7), 1665–1671 (1999). 10423134 10.1099/0022-1317-80-7-1665

[b24] Axten J. M. *et al.* Discovery of 7-methyl-5-(1-{[3-(trifluoromethyl)phenyl]acetyl}-2,3-dihydro-1H-indol-5-yl)-7H-p yrrolo[2,3-d]pyrimidin-4-amine (GSK2606414), a potent and selective first-in-class inhibitor of protein kinase R (PKR)-like endoplasmic reticulum kinase (PERK). J Med Chem 55, 7193–7207 (2012). 22827572 10.1021/jm300713s

[b25] Yoshida H., Matsui T., Yamamoto A., Okada T. & Mori K. XBP1 mRNA is induced by ATF6 and spliced by IRE1 in response to ER stress to produce a highly active transcription factor. Cell 107, 881–891 (2001). 11779464 10.1016/s0092-8674(01)00611-0

[b26] Papandreou I. *et al.* Identification of an Ire1alpha endonuclease specific inhibitor with cytotoxic activity against human multiple myeloma. Blood 117, 1311–1314 (2011). 21081713 10.1182/blood-2010-08-303099 PMC3056474

[b27] Bhattacharyya S., Sen U. & Vrati S. Regulated IRE1-dependent decay pathway is activated during Japanese encephalitis virus-induced unfolded protein response and benefits viral replication. J Gen Virol 95, 71–79 (2014). 24114795 10.1099/vir.0.057265-0

[b28] Ma Y. & Hendershot L. M. Delineation of a negative feedback regulatory loop that controls protein translation during endoplasmic reticulum stress. J Biol Chem 278, 34864–34873 (2003). 12840028 10.1074/jbc.M301107200

[b29] Stockwell S. R. *et al.* Mechanism-based screen for G1/S checkpoint activators identifies a selective activator of EIF2AK3/PERK signalling. PLoS One 7, e28568 (2012). 22253692 10.1371/journal.pone.0028568 PMC3257223

[b30] Chitnis N., Pytel D. & Diehl J. A. UPR-inducible miRNAs contribute to stressful situations. Trends Biochem Sci 38, 447–452 (2013). 23906563 10.1016/j.tibs.2013.06.012 PMC4056666

[b31] Leucci E. *et al.* microRNA-9 targets the long non-coding RNA *MALAT1* for degradation in the nucleus. Sci Rep 3, 2535 (2013). 23985560 10.1038/srep02535 PMC3756333

[b32] Wang X. *et al.* Silencing of long noncoding RNA *MALAT1* by miR-101 and miR-217 inhibits proliferation, migration, and invasion of esophageal squamous cell carcinoma cells. J Biol Chem 290, 3925–3935 (2015). 25538231 10.1074/jbc.M114.596866 PMC4326802

[b33] Lee A. H., Iwakoshi N. N. & Glimcher L. H. XBP-1 regulates a subset of endoplasmic reticulum resident chaperone genes in the unfolded protein response. Mol Cell Biol 23, 7448–7459 (2003). 14559994 10.1128/MCB.23.21.7448-7459.2003 PMC207643

[b34] Averous J. *et al.* Induction of CHOP expression by amino acid limitation requires both ATF4 expression and ATF2 phosphorylation. J Biol Chem 279, 5288–5297 (2004). 14630918 10.1074/jbc.M311862200

[b35] Hollien J. *et al.* Regulated Ire1-dependent decay of messenger RNAs in mammalian cells. J Cell Biol 186, 323–331 (2009). 19651891 10.1083/jcb.200903014 PMC2728407

[b36] Domingo E., Sheldon J. & Perales C. Viral quasispecies evolution. Microbiol Mol Biol Rev 76, 159–216 (2012). 22688811 10.1128/MMBR.05023-11 PMC3372249

[b37] Hu L. *et al.* Up-regulation of long noncoding RNA *MALAT1* contributes to proliferation and metastasis in esophageal squamous cell carcinoma. J Exp Clin Cancer Res 34, 7 (2015). 25613496 10.1186/s13046-015-0123-z PMC4322446

[b38] Michalik K. M. *et al.* Long noncoding RNA *MALAT1* regulates endothelial cell function and vessel growth. Circ Res 114, 1389–1397 (2014). 24602777 10.1161/CIRCRESAHA.114.303265

[b39] Bi M. *et al.* ER stress-regulated translation increases tolerance to extreme hypoxia and promotes tumor growth. Embo J 24, 3470–3481 (2005). 16148948 10.1038/sj.emboj.7600777 PMC1276162

[b40] Fan Y. *et al.* TGF-beta-induced upregulation of *malat1* promotes bladder cancer metastasis by associating with suz12. Clin Cancer Res 20, 1531–1541 (2014). 24449823 10.1158/1078-0432.CCR-13-1455

[b41] Li S. *et al.* Sp1-mediated transcriptional regulation of *MALAT1* plays a critical role in tumor. J Cancer Res Clin Oncol (2015). 10.1007/s00432-015-1951-0 PMC11824113 25773124

[b42] Gutschner T. *et al.* The noncoding RNA *MALAT1* is a critical regulator of the metastasis phenotype of lung cancer cells. Cancer Res 73, 1180–1189 (2013). 23243023 10.1158/0008-5472.CAN-12-2850 PMC3589741

[b43] Miyagawa R. *et al.* Identification of cis- and trans-acting factors involved in the localization of MALAT-1 noncoding RNA to nuclear speckles. Rna 18, 738–751 (2012). 22355166 10.1261/rna.028639.111 PMC3312561

[b44] Nakagawa S. *et al.* *Malat1* is not an essential component of nuclear speckles in mice. Rna 18, 1487–1499 (2012). 22718948 10.1261/rna.033217.112 PMC3404370

[b45] Engreitz J. M. *et al.* RNA-RNA interactions enable specific targeting of noncoding RNAs to nascent Pre-mRNAs and chromatin sites. Cell 159, 188–199 (2014). 25259926 10.1016/j.cell.2014.08.018 PMC4177037

[b46] Dong Y. *et al.* *MALAT1* promotes the proliferation and metastasis of osteosarcoma cells by activating the PI3K/Akt pathway. Tumour Biol 36, 1477–1486 (2015). 25431257 10.1007/s13277-014-2631-4

[b47] Wu X. S. *et al.* *MALAT1* promotes the proliferation and metastasis of gallbladder cancer cells by activating the ERK/MAPK pathway. Cancer Biol Ther 15, 806–814 (2014). 24658096 10.4161/cbt.28584 PMC4049796

[b48] Puthanveetil P., Chen S., Feng B., Gautam A. & Chakrabarti S. Long non-coding RNA *MALAT1* regulates hyperglycaemia induced inflammatory process in the endothelial cells. J Cell Mol Med 19, 1418–1425 (2015). 25787249 10.1111/jcmm.12576 PMC4459855

[b49] Han X., Yang F., Cao H. & Liang Z. *Malat1* regulates serum response factor through miR-133 as a competing endogenous RNA in myogenesis. Faseb J (2015). 10.1096/fj.14-259952 25868726

[b50] Liu Y. P. *et al.* Endoplasmic reticulum stress regulates the innate immunity critical transcription factor IRF3. J Immunol 189, 4630–4639 (2012). 23028052 10.4049/jimmunol.1102737 PMC3478468

[b51] Saha S., Murthy S. & Rangarajan P. N. Identification and characterization of a virus-inducible non-coding RNA in mouse brain. J Gen Virol 87, 1991–1995 (2006). 16760401 10.1099/vir.0.81768-0

[b52] Jiang Y. *et al.* The role of *MALAT1* correlates with HPV in cervical cancer. Oncol Lett 7, 2135–2141 (2014). 24932303 10.3892/ol.2014.1996 PMC4049771

[b53] Zhang Q., Chen C. Y., Yedavalli V. S. & Jeang K. T. NEAT1 long noncoding RNA and paraspeckle bodies modulate HIV-1 posttranscriptional expression. MBio 4, e00596–00512 (2013). 23362321 10.1128/mBio.00596-12 PMC3560530

